# *N*-carboxyacyl and *N*-α-aminoacyl derivatives of aminoaldehydes as shared substrates of plant aldehyde dehydrogenases 10 and 7

**DOI:** 10.1007/s00726-024-03415-4

**Published:** 2024-08-29

**Authors:** Michaela Masopustová, Adam Goga, Miroslav Soural, Martina Kopečná, Marek Šebela

**Affiliations:** 1 Department of Biochemistry, Faculty of Science, Palacký University, Olomouc, Czech Republic; 2 Department of Organic Chemistry, Faculty of Science, Palacký University, Olomouc, Czech Republic; 3 Department of Experimental Biology, Faculty of Science, Palacký University, Olomouc, Czech Republic

**Keywords:** Acylation, Aminoaldehyde, Aldehyde dehydrogenase, Docking, Enzyme, Substrate

## Abstract

Aldehyde dehydrogenases (ALDHs) represent a superfamily of enzymes, which oxidize aldehydes to the corresponding acids. Certain families, namely ALDH9 and ALDH10, are best active with ω-aminoaldehydes arising from the metabolism of polyamines such as 3-aminopropionaldehyde and 4-aminobutyraldehyde. Plant ALDH10s show broad specificity and accept many different aldehydes (aliphatic, aromatic and heterocyclic) as substrates. This work involved the above-mentioned aminoaldehydes acylated with dicarboxylic acids, phenylalanine, and tyrosine. The resulting products were then examined with native ALDH10 from pea and recombinant ALDH7s from pea and maize. This investigation aimed to find a common efficient substrate for the two plant ALDH families. One of the best natural substrates of ALDH7s is aminoadipic semialdehyde carrying a carboxylic group opposite the aldehyde group. The substrate properties of the new compounds were demonstrated by mass spectrometry of the reaction mixtures, spectrophotometric assays and molecular docking. The *N*-carboxyacyl derivatives were good substrates of pea ALDH10 but were only weakly oxidized by the two plant ALDH7s. The *N*-phenylalanyl and *N*-tyrosyl derivatives of 3-aminopropionaldehyde were good substrates of pea and maize ALDH7. Particularly the former compound was converted very efficiently (based on the *k*_cat_/*K*_m_ ratio), but it was only weakly oxidized by pea ALDH10. Although no compound exhibited the same level of substrate properties for both ALDH families, we show that these enzymes may possess more common substrates than expected.

**Supplementary Information:**

The online version contains supplementary material available at 10.1007/s00726-024-03415-4.

## Introduction

Aldehydes are reactive compounds, which are eliminated from the cellular environment to prevent biological molecules from damaging by unwanted modifications (O'Brien et al. 2005). There is a superfamily of polymorphic enzymes, aldehyde dehydrogenases (ALDHs), which are responsible for the metabolic conversion of endo- as well as exogenous aldehydes to less harmful acids (Koppaka et al. 2012). The superfamily consists of 27 families (Riveros-Rosas et al. 2013) based on the level of amino acid sequence identity. In plants, there are 14 distinct ALDH families numbered 2, 3, 5–7, 10–12, 18, 19, 21–24, from which only the ALDHs 10, 12, 19, 21–24 are specific to plants (Hou and Barthels 2015; Brocker et al. 2013). Sometimes, the family ALDH19 is not included as it has been reported only in tomatoes among higher plants (Jimenez-Lopez et al. 2016).

ALDHs are oligomeric enzymes, typically existing as dimers or tetramers. Their subunits comprise three major functional domains: a catalytic, a coenzyme-binding, and an oligomerization domain and they utilize NAD^+^ or NADP^+^ as coenzymes. The active site is located at the interface of the catalytic and coenzyme-binding domains. It is largely conserved throughout the superfamily (Koppaka et al. 2012). In the catalytic process of pea seedling aminoaldehyde dehydrogenase isoenzymes (PsAMADH1 and 2) from the ALDH10 family, Cys294 and Glu260 play crucial roles as the most important amino acid residues, complemented by Asn162 (Tylichová et al. 2010). Cys294 serves as the active-site nucleophile, Glu260 acts as the general base with a proton abstraction role in the catalysis, and Asn162 stabilizes the thiohemiacetal and thioester reaction intermediates through hydrogen bonds. All these residues are situated at the narrowed bottom of a substrate channel extending from the enzyme's surface (Kopečný et al. 2013). Similarly, a catalytic cysteine is present at the active site of ALDH7s (Tang et al. 2008; Luo and Tanner 2015), specifically Cys301 in the maize enzyme ZmALDH7 (Končitíková et al. 2015). Other important residues conserved among ALDH7s include Glu120, Arg300, and Thr302 (in ZmALDH7 numbering). The former two form ion pairs that bind to the amino and carboxylic groups, respectively, of the most preferred substrate l-α-aminoadipate-δ-semialdehyde (AASAL) within lysine metabolism. The methyl ester of adipic semialdehyde represents another good substrate for ZmALDH7 (Končitíková et al. 2015) with a comparable binding affinity but a much lower oxidation rate.

Enzymes from the family ALDH10 are recognized for their ability to oxidize various natural ω-aminoaldehydes. Examples of these include 3-aminopropionaldehyde (APAL), 4-aminobutyraldehyde (ABAL), 4-guanidinobutyraldehyde, betaine aldehyde and 4-(trimethylamino)butyraldehyde, all of which are produced in the metabolism of amino acids and polyamines (Brocker et al. 2013). Plant ALDH10s have demonstrated a broad substrate specificity, attributed to the funnel-shaped substrate channel and its amino acid composition at the entrance and upper part (Kopečný et al. 2013). Additionally, these enzymes convert various synthetic aminoaldehyde and aldehyde substrates, including pyridine carbaldehydes, pyridinyl propanals, pyridinylmethylamino propanals, bromobenzaldehydes and aminoaldehydes with a purine, 7-deazapurine or pyrimidine ring (Frömmel et al. 2012). *N*-acylated aminoaldehydes are also good synthetic substrates (Frömmel et al. 2015), while aldehydes containing imidazole or pyrazole are only weakly oxidized (Frömmel et al. 2019). Plant ALDH7 enzymes also oxidize the aforementioned natural aminoaldehydes, albeit weakly compared to the best substrate, AASAL (Končitíková et al. 2015).

The availability of substrates accepted by enzymes from a specific ALDH family allows their application in fundamental and comparative biochemical research, including kinetic measurements and activity-based staining after electrophoretic separations or in situ in tissues during microscopy (Šebela et al. 2001). Recently, the PauC ALDH enzyme from *Pseudomonas aeruginosa*, acting in the polyamine breakdown pathway, was found to efficiently oxidize 3-[(l-γ-glutamyl)amino]propionaldehyde (produced from glutamylated polyamines) more than APAL (Cardona-Cardona et al. 2022). This suggests that the latter may accumulate and exert toxicity when the enzyme's activity is low or inhibited.

To our knowledge, there is no common substrate with a comparable binding and turnover rate available for the enzymes of the ALDH families 7, 9, and 10. This limits the ability to perform kinetic measurements of the total production rate of compatible osmolytes in tissue extracts. In this study, APAL and ABAL were acylated by dicarboxylic acids as *N*-adipoyl-APAL has been shown to be a good substrate for PsAMADH1 and 2, with *k*_cat_ of 8.8 s^−1^, and *K*_m_ of 430 and 40 μmol·l^−1^, respectively (Frömmel et al. 2015). The resulting *N*-carboxyacyl derivatives, carrying a negative charge opposite the aldehyde group, were analyzed as substrates for both native PsAMADH and recombinant ZmALDH7 and PsALDH7. The ω-amino group of the parental compounds, protonated at physiological pH, is replaced by an uncharged amido group, resulting in a charge inversion of the molecule due to acylation. *N*-α-Phenylalanyl and *N*-α-tyrosyl derivatives of APAL and ABAL were prepared to introduce an aromatic ring at the terminus opposite the aldehyde group. Studying enzyme activities with synthetic substrates provides a powerful and versatile toolset for researchers to gain a deep understanding of enzymatic mechanisms, substrate specificity, and kinetic properties. The importance of the chain length, presence of terminal carboxylate, or aromatic ring on the substrate properties was characterized. Additionally, the differences between plant ALDH10s and ALDH7s in the kinetic parameters were examined.

## Materials and methods

### Chemicals and other materials

Alpha-cyano-4-hydroxycinnamic acid (CHCA) was purchased from Bruker Daltonik (Bremen, Germany). APAL and ABAL diethylacetals, *N*,*N*-diisopropylethylamine (DIPEA) and β-nicotinamide adenine dinucleotide (NAD^+^) sodium salt were purchased from Sigma-Aldrich Chemie (Steinheim, Germany). Monoethylester chlorides of malonic, succinic and glutaric acids were from the same vendor. l-α-Aminoadipate-δ-semialdehyde (AASAL) ethyleneacetal was obtained from Chiralix (Nijmegen, the Netherlands). *N*-(*tert*-Butoxycarbonyl)-l-phenylalanine (Boc-Phe-OH) and *N*-(*tert*-butoxycarbonyl)-O-*tert*-butyl-l-tyrosine [Boc-Tyr(*t*Bu)-OH] were purchased from Fluorochem (Hadfield, United Kingdom). All other chemicals were of analytical purity grade. Cetyltrimethylammonium bromide (CTAB), analytical quality grade, was provided by Prof. Juraj Ševčík, Faculty of Science, Palacký University.

### Enzymes

Native pea aminoaldehyde dehydrogenase (PsAMADH, a mixture with prevailing isoenzyme 1), belonging to the class ALDH10, was purified from etiolated pea seedlings as described (Šebela et al. 2000) with several modifications. The first chromatographic step on Octyl-Sepharose was omitted, DEAE-cellulose SH-23 was replaced by DEAE-Sepharose, and the gel chromatography step was conducted on an ENrich SEC 650 column (Bio-Rad, Hercules, CA, USA) instead of Sephacryl S-300 HR. Pea and maize ALDH7 (PsALDH7 and ZmALDH7, respectively) were obtained as recombinant proteins (Končitíková et al. 2015). The specific activities with reference substrates (1 mmol·l^−1^) were as follows: 30–40 nkat·mg^−1^ (PsAMADH with APAL; different isolates), 10 nkat·mg^−1^ (PsALDH7 with AASAL) and 11 nkat·mg^−1^ (ZmALDH7 with AASAL). The purity of the enzymes was analyzed by gel electrophoresis and tandem mass spectrometry of peptides in tryptic digests as documented in Supplementary file 1, where also a multiple sequence alignment is included.

### Synthesis of *N*-acylated aminoaldehydes

**Fig. 1:**
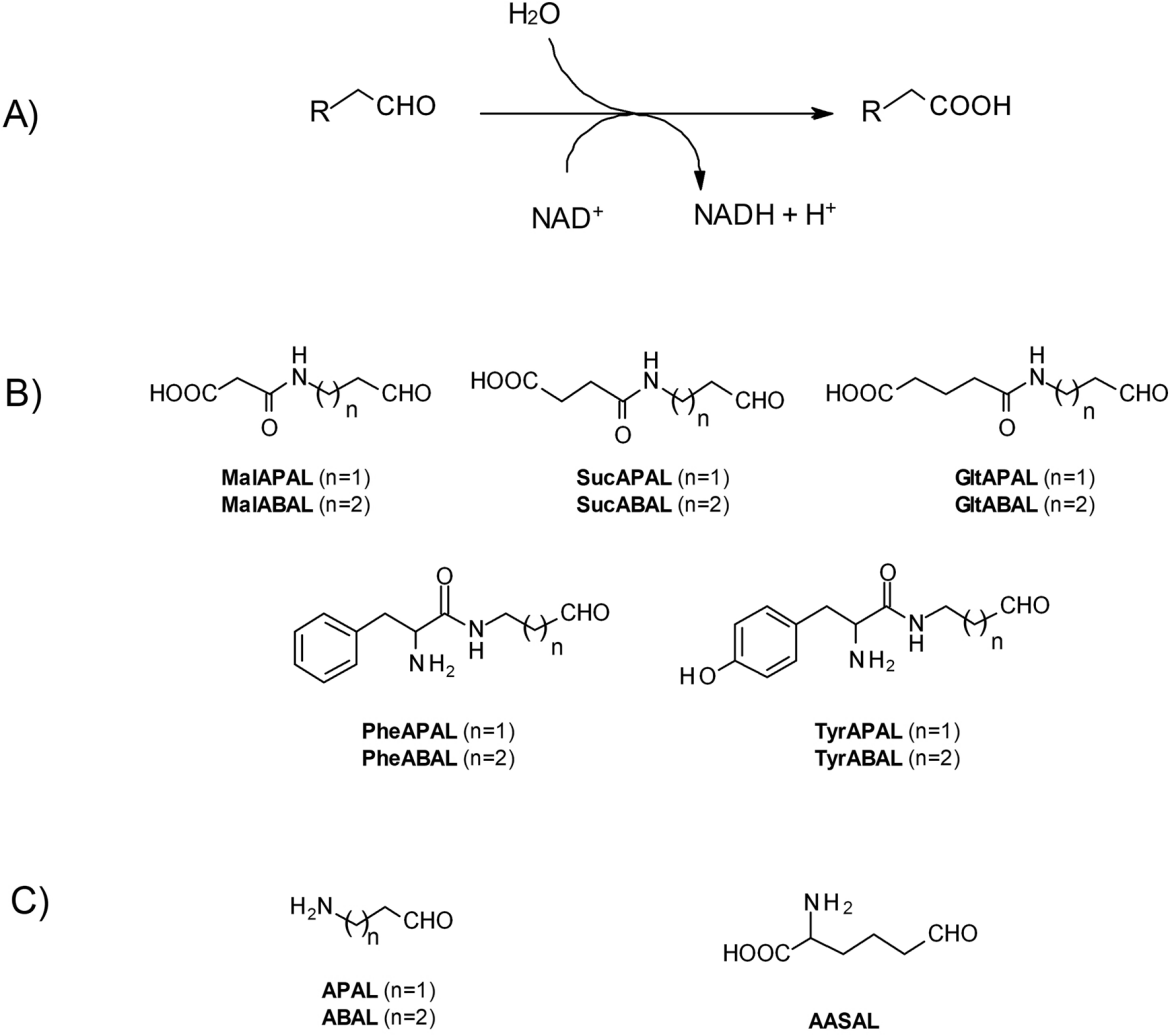
The reaction of ALDH10/ALDH7 and the chemical structures of the studied *N*-carboxyacyl and *N*-α-aminoacyl APAL/ABAL derivatives. **A** Shows a general scheme of the reaction catalyzed by ALDH10/ALDH7; **B** summarizes the chemical formulas of all novel synthetic substrates, and **C** illustrates the natural substrates used as references in the kinetic experiments. All abbreviations are explained in the List of Abbreviations

The chemical formulas of all synthesized compounds are shown in Fig. 1 together with their name abbreviations. The identity and purity of synthesis products was evaluated by NMR performed using ECA400II and ECX500 spectrometer (JEOL RESONANCE, Tokyo, Japan) at magnetic field strength of 9.39 T and 11.75 T corresponding to ^1^H and ^13^C resonance frequencies of 399.78 MHz and 500.16 MHz, and 100.53 MHz and 125.77 MHz at 27 °C. Chemical shifts (*δ*) are reported in *parts per million* (ppm) and coupling constants (*J*) are reported in Hertz (Hz); all NMR data are provided in Supplementary file 1.

APAL or ABAL diethylacetal (6 mmol) was mixed with 50 ml of dry dichloromethane and DIPEA (7 mmol) was added. The mixture was kept at laboratory temperature under continuous stirring on a magnetic stirrer for 15 min. The respective dicarboxylic acid monoethylester chloride (6 mmol) was then added dropwise over a period of 30 min. The crude product solution was set aside with stirring for an additional time period of 30 min. This was followed by consecutive extractions with 10% acetic acid, 10% sodium bicarbonate and water in a separating funnel to remove salts. The organic layer was dried over sodium sulfate at 4 °C overnight, filtered and the solvent removed using a rotary vacuum evaporator to yield the final product as a yellow oil.

Boc-Phe-OH or Boc-Tyr(*t*Bu)-OH (6 mmol) and 1,3-dicyclohexylcarbodiimide (3 mmol) were dissolved together in 50 ml of dichloromethane under a continuous stirring. A white precipitate formed within a few minutes and was filtered off. Then ABAL or APAL (6 mmol) was added by a pipette in three aliquots with 5-min waiting periods. The mixture was stirred for additional 30 min and filtered. Then it was sequentially shaken with 50 ml of 10% acetic acid, 50 ml water, 50 ml of 5% sodium bicarbonate and 50 ml water in a separating funnel. The organic layer was recovered and finally dried over sodium sulfate. White solid products were obtained on evaporating the solvent.

### Hydrolysis of diethylacetals/protective groups and aminoaldehyde analysis by mass spectrometry

Diethylacetals were converted to deprotected aldehydes by heating their aliquots (10–20 mg) with 0.5 ml of 0.5 mol·l^−1^ HCl in plugged test tubes at 100 °C for 15 min [APAL derivatives, Boc-PheABAL, Boc-Tyr(tBu)ABAL], and at 70 °C for 2 h (all other ABAL derivatives). The dissolution of Boc-Tyr(*t*Bu)APAL and Boc-Tyr(*t*Bu)ABAL diethylacetals was facilitated by adding 50 µl methanol prior to the addition of 0.5 mol·l^−1^ HCl. AASAL ethyleneacetal, 18.9 mg, was dissolved in 0.6 ml of 0.5 mol·l^−1^ HCl and heated at 100 °C for 15 min. The pH of the solutions was adjusted to a less acidic value of around pH 4 by adding 25% NH_4_OH (approximately 20 µl), confirmed using narrow indicator paper strips. The final solution volume was adjusted to 1 ml with deionized water.

The synthesized acylated amidoaldehydes were analyzed on a Microflex LRF 20 MALDI-TOF (matrix-assisted laser desorption/ionization time-of-flight) mass spectrometer equipped with a 60-Hz nitrogen laser operating at a maximum wavelength of 337 nm (Bruker Daltonik). CHCA was used as a matrix in the presence of CTAB following the method described by Guo et al. 2002. To prepare the matrix solution, CHCA was dissolved to a concentration of 0.1 mol·l^−1^ in a mixture of water and acetone (1:4, v/v). This solution (1 ml) was then supplemented with CTAB (10 mmol·l^−1^ in the same solvent, 10 μl) to achieve a molar ratio of CHCA-to-CTAB of 1000:1. Free aminoaldehyde samples were measured as 1 mmol·l^−1^ solutions. The experimental procedure involved depositing 1 μl of the matrix solution onto the target plate and allowing it to dry in the air, resulting in a thin layer of matrix crystals. Subsequently, 1 μl of the sample was applied on the matrix layer and left to dry. Mass spectra acquisition was carried out in the reflector positive ion mode using the following parameter settings: 1) acceleration voltage (IS1) of 19.0 kV, extraction voltage (IS2) of 16.2 kV, lens voltage of 8.9 kV, reflector voltage of 20.0 kV, detector voltage of 1670 V and pulsed-ion extraction delay time of 150 ns. Mass spectra were acquired in the *m/z* range of 0–2000, and internal calibration was performed using specific CHCA matrix ions (*m/z* 190.05 and 379.09).

### Enzyme activity assays

ALDH10 activity was assayed spectrophotometrically at 340 nm by monitoring the formation of NADH (ε_340_ = 6.22 mmol^−1^·l·cm^−1^) produced during the substrate oxidation. The instrument used was a Lightwave II UV–Vis spectrophotometer (Biochrom, Cambridge, UK) underlaid with an electromagnetic stirring plate IKA lab disc (IKA, Staufen, Germany). The reaction mixture for ALDH10 (2 ml) in a 3-ml cuvette, continuously stirred, contained 0.15 mol·l^−1^ Tris–HCl, pH 9.0, 1 mmol·l^−1^ NAD^+^, enzyme solution (10–15 μl) and the measured substrate in micromolar to millimolar concentrations for determining the kinetic parameters *k*_cat_ and *K*_m_. The substrate was always added as the final component to initiate the reaction. The duration of the measurements was 3 min with recording absorbance values each 30 s to check the linearity of the time-dependent increase in absorption. In the case of ALDH7, the reaction buffer was 0.1 mol·l^−1^ sodium pyrophosphate, pH 8.1, and the concentration of NAD^+^ was increased to 2.5 mmol·l^−1^. All individual measurements were performed in triplicates. Data from kinetic measurements were processed using Graph Pad Prism 8.0.1 software.

### Reaction product analysis

The reaction mixture, with a final volume of 2 ml, was prepared by combining 1790 µl of 112 mmol·l^−1^ NH_4_HCO_3_, 2 µl of 25% (w/v) ammonia, 50 µl of 40 mmol·l^−1^ NAD^+^, and 143 µl containing water and the respective substrate after ester-acetal hydrolysis (the final substrate concentration was 1 mmol·l^−1^). Substrate conversion was initiated by adding 15 µl of the enzyme solution, and the entire mixture was then incubated at 37 °C overnight. *N*-α-Phenylalanyl and *N*-α-tyrosyl derivatives of APAL and ABAL were processed as follows: 1 mL of 50 mmol·l^−1^ NH_4_HCO_3_ was mixed with 30 µL of 30 mmol·l^−1^ aminoaldehyde (a fresh neutralized solution after acetal hydrolysis), and 25 µL of 40 mmol·l^−1^ NAD^+^. The reaction was initiated by adding 10 µL of enzyme and proceeded at 37 °C for 1 h. Analyses of the product acids were generally performed by MALDI-TOF MS as described above. Aliquots of 1 µl were taken from the reaction mixtures and deposited on the target plate with the layer of matrix crystals.

### Molecular docking

Aldehyde ligand topologies for molecular docking were obtained as PDB-formatted files using the PRODRG server operated at the University of Dundee (Schüttelkopf and van Aalten 2004). Protein coordinates (PsAMADH1, accession code: 3IWK; PsAMADH2, accession code: 3IWJ; ZmALDH7, accession code: 4PXN) were downloaded from the PDB database at https://www.rcsb.org/pdb. Docking of the ligands into ALDH structures was performed via the Achilles Blind Docking Server at the Catholic University of Murcia (http://bio-hpc.eu/software/blind-docking-server/, Sánchez-Linares et al. 2012).

## Results

### Characterization of synthesized compounds by instrumental analysis

All compounds subjected to this study (Fig. 1) were synthesized in the form of diethylacetals. In addition, the products contained other protecting groups—ethyl ester, *tert*-butyloxycarbonyl (Boc) and *tert*-butyl (*t*Bu), which protected carboxyl groups, amino groups and tyrosine hydroxyls, respectively. The chemical identity was confirmed by measuring ^1^H- and ^13^C-NMR spectra as shown in Supplementary file 1.

**Fig. 2:**
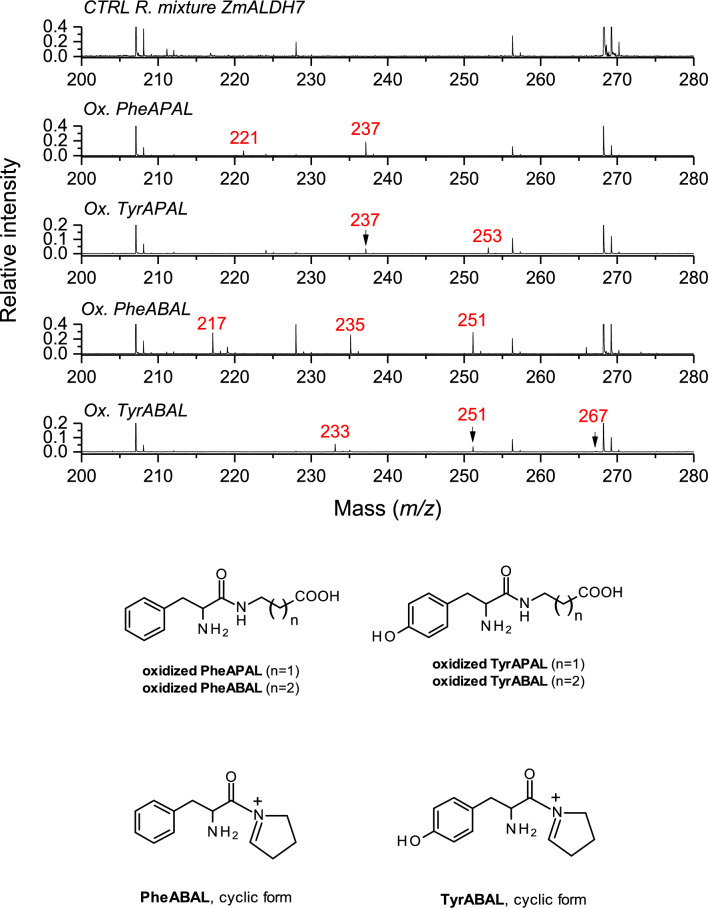
MALDI-TOF mass spectra of ZmALDH7 reaction mixtures. PheAPAL, TyrAPAL, PheABAL and TyrABAL (1 mM; as indicated by spectrum labeling) were oxidized by the enzyme in 50 mmol·l^−1^ NH_4_HCO_3_ for 1 h. The corresponding product carboxylic acids (the chemical formulas are shown at the bottom) provided peaks with *m/z* values of 237, 253, 251 and 267, respectively. The residual unreacted aldehyde substrates were detectable at *m/z* 221, 237, 235 and 251, respectively. PheABAL and TyrABAL could be detected also in their cyclic forms (*m/z* 217 and 233, respectively); the chemical formulas are shown at the bottom as well. The spectra were measured using CHCA matrix in the presence of CTAB. The control spectrum shows matrix-related background signals

MALDI-TOF MS in the positive ion mode was used to evaluate the cleavage of acetals and protecting groups. The complete hydrolysis and deprotection for the ethylester-acetal derivatives of APAL was achieved by incubating with diluted hydrochloric acid at 100 °C for 15 min. A prolonged incubation at 70 °C for 2 h was found necessary for the corresponding ABAL-derived compounds. The pseudomolecular ions [M + H]^+^ of free MalAPAL (*m/z* 160), SucAPAL (*m/z* 174), and GltAPAL (*m/z* 188) confirmed the loss of ethanol moieties by hydrolysis of the acetal as well as ethylester groups (Supplementary file 2). The free MalABAL, SucABAL and GltABAL (*m/z* 174, 188 and 202, respectively) were found to partially cyclize after the acid hydrolysis as the experimental masses were reduced by 18 Da contrary to theoretical expectations (*m/z* 156, 170 and 184, respectively). Incomplete hydrolysis (i.e. cleavage of acetals but not ethylesters) was observed when acquiring mass spectra immediately after crystallization with matrix solution without any preceding thermal treatment in a strongly acidic environment (Supplementary file 2). For example, the *m/z* value of 198 registered for direct measurements with SucABAL diethylacetal corresponds to a cyclized (i.e. *Δ*^1^-pyrrolinium) form of SucABAL still bearing the ester group, whereas *m/z* 244 refers to a loss of ethanol (*m/z* difference of 46) from the original diethylacetal molecule. The free aldehydes of PheAPAL, TyrAPAL, PheABAL, and TyrABAL provided pseudomolecular ion [M + H]^+^ peaks with *m/z* values of 221, 237, 235, and 251, respectively, in accordance with their theoretical molecular masses (Fig. 2). The ABAL derivatives additionally showed a partial presence of the corresponding cyclic (pyrrolinium) forms with *m/z* 217 and 233 (PheABAL and TyrABAL, respectively), as seen in Fig. 2.

### Detection of reaction products in reaction mixtures

The synthetic compounds were characterized in reactions with three different enzymes, namely native PsAMADH and recombinant ALDH7s from pea and maize. The native enzyme was isolated from 7-day-old pea seedlings and represented a mixture of both isoenzymes PsAMADH1 and 2.

MALDI-TOF MS measurements were used to initially analyze substrate conversion. These assays involved incubating the compounds with the enzymes in 50 or 100 mM NH_4_HCO_3_ serving as a volatile buffer. For compounds acylated by dicarboxylic acids, clear analysis of conversion was possible only for GltAPAL and GltABAL. The matrix system used caused peak interference, hindering unambiguous detection of product signals for other compounds. In the positive ion mode, the oxidized GltAPAL and GltABAL provided product peaks with *m/z* 204 and 218, respectively. These values corresponded to the theoretical expectations for 5-[(2-carboxyethyl)amino]-5-oxopentanoic acid and 5-[(3-carboxypropyl)amino]-5-oxopentanoic acid, respectively (Supplementary file 2). However, the recorded signal intensity was low, especially for oxidized GltAPAL. For Phe- and Tyr-based derivatives, oxidation product peaks were observed at *m/z* 237 (oxidized PheAPAL), 253 (oxidized TyrAPAL), 251 (oxidized PheABAL), and 267 (oxidized TyrABAL). These values align with the expected chemical structures of the corresponding carboxylic acids: 3-[(2-amino-3-phenylpropanoyl)amino]propanoic acid, 3-{[2-amino-3-(4-hydroxyphenyl)propanoyl]amino}propanoic acid, 4-[(2-amino-3-phenylpropanoyl)amino]butanoic acid, and 4-[2-amino-3-(4-hydroxyphenyl)propanoyl]amino}butanoic acid, respectively, as illustrated in Fig. 2. The oxidation product of TyrABAL showed a very low intensity signal compared to the oxidation of PheAPAL and PheABAL.

### Spectrophotometric assays to determine kinetic parameters of enzymatic reactions

The initial orientation measurements were conducted using 1 mmol·l^−1^ compounds in the reaction mixture, with pH adjusted to 9.0 for PsAMADH and 8.1 for ALDH7s. This concentration was chosen to ensure saturation, relying on previously published data with other substrates (Tylichová et al. 2010; Končitíková et al. 2015). NADH production was monitored at 340 nm, and the corresponding enzymatic activity was calculated from the slopes of the time-dependent absorbance curves. The first group of synthesized compounds, derived from dicarboxylic acids, exhibited excellent substrate properties for PsAMADH, with observed relative initial reaction rates between 40 and 60% compared to APAL (Supplementary file 2). Notably, SucAPAL showed an even higher rate of 110%. For PsALDH7 and ZmALDH7, the relative reaction rates were approximately 2% compared to the best substrate AASAL, with the exception of MalAPAL/MalABAL (5–6%). The second group of new substrates, derived from the amino acids Phe and Tyr, exhibited significantly lower relative reaction rates with PsAMADH (3–6%). However, PheAPAL and TyrAPAL were found good substrates for the two ALDH7s (8–12% compared to AASAL), whereas PheABAL and TyrABAL were oxidized at lower relative rates below 5% (Supplementary file 2).

**Fig. 3:**
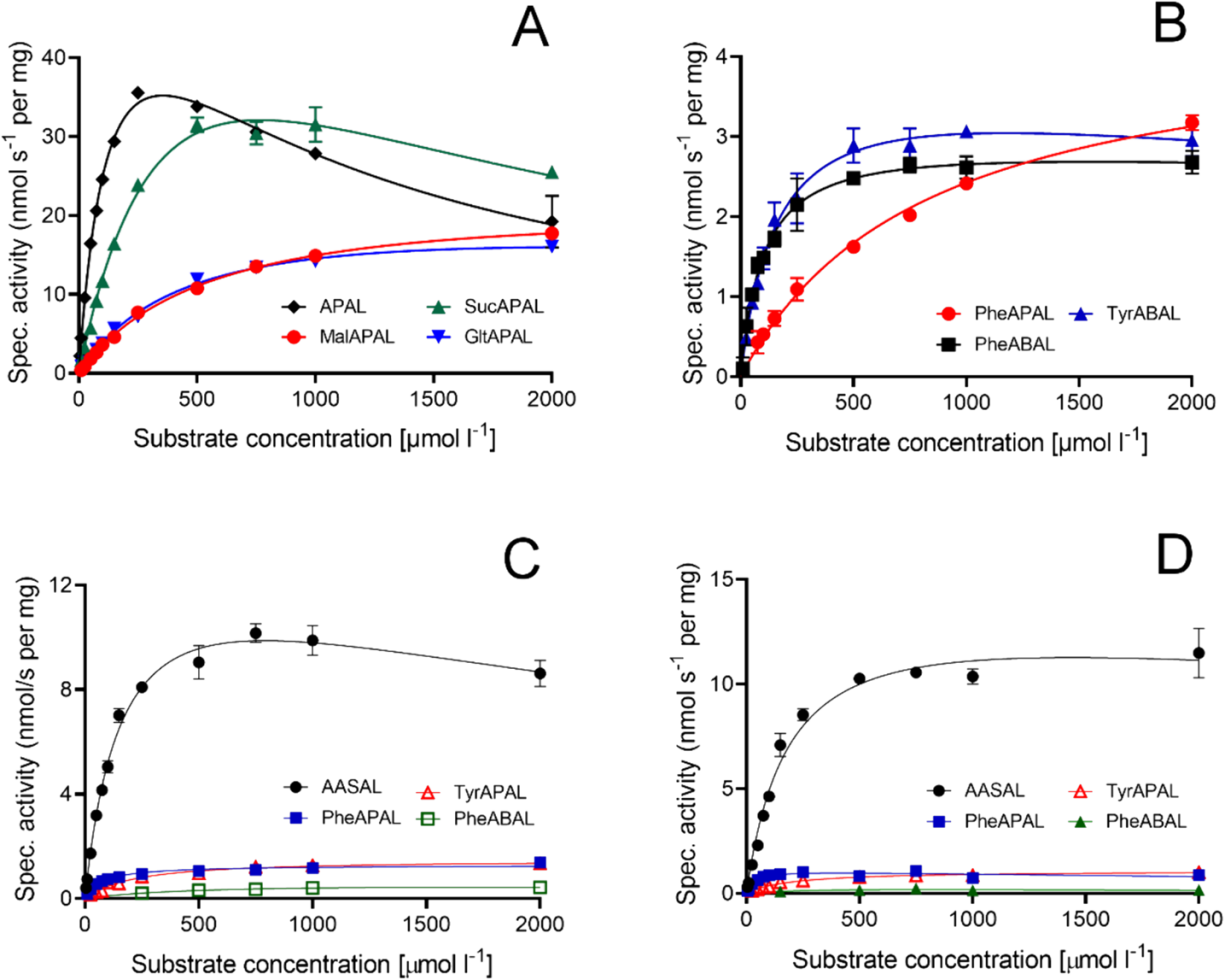
Saturation curves of native PsAMADH reactions with synthetic *N*-carboxyacyl and *N*-α-aminoacyl aminoaldehydes, and PsALDH7/ZmALDH7 reactions with *N*-α-aminoacyl aminoaldehydes. All PsAMADH measurements were conducted at pH 9.0 with the initial presence of 1 mmol·l^−1^ NAD^+^ and varying substrate concentrations. For the two ALDHs, the pH value was set at 8.1 and the NAD^+^ concentration was increased to 2.5 mmol·l^−1^. The depicted data were averaged from three independent measurements, as indicated by error bars. Individual substrates are represented by color coding, as explained at each graph. **A** PsAMADH; **B** PsAMADH; **C** PsALDH7; **D** ZmALDH7. APAL and AASAL were reference substrates for PsAMADH and ALDH7s, respectively

The *N*-carboxyacyl aminoaldehydes were analyzed in measurements with native PsAMADH to determine the kinetic parameters *k*_cat_ and *K*_m_. This analysis utilized spectrophotometry by monitoring NADH production in the reaction mixture at 340 nm (illustrated in Fig. 3 and Supplementary file 2). The measured *K*_m_ values were predominantly around 500 µmol·l^−1^, except for MalABAL (879 µmol·l^−1^) and SucABAL (138 µmol·l^−1^). The *k*_cat_ values ranged between 1.3 and 1.6 s^−1^, only SucAPAL exhibited a relatively high value of 4.1 s^−1^ (Table 1). Two compounds in the series, SucABAL and SucAPAL, demonstrated substrate properties comparable to the natural substrates, yielding relative efficiency constant values of 0.474 and 0.328, respectively, compared to APAL (set as a reference at 1) and ABAL (0.526); see in Table 1. The excess substrate inhibition constants determined with SucAPAL (1.2 mmol·l^−1^) and SucABAL (1.4 mmol·l^−1^) corresponded with that for APAL (1.1 mmol·l^−1^). Parallel measurements with ALDH7s were not conducted due to low activity at less than millimolar substrate concentrations. Only the kinetic parameters for MalAPAL and MalABAL could be determined, but the corresponding relative efficiency constants are relatively low compared to AASAL because of the high *K*_m_ values (Table 2). The second substrate group, *N*-aminoacyl aminoaldehydes, was subjected to kinetic measurements with PsAMADH, PsALDH7 and ZmALDH7 too (Fig. 3). With PsAMADH, the kinetic constants were obtained for PheAPAL, PheABAL and TyrABAL (Table 3). The *K*_m_ values ranged between 100 and 820 µmol·l^−1^ and the *k*_cat_ values were around 0.2 s^−1^. PheABAL emerged as the best substrate from this group with a relative catalytic efficiency of 0.05 compared to the natural substrate APAL. The two ALDH7s showed *K*_m_ values of 200–660 µmol·l^−1^ for PheABAL and TyrAPAL, while PheAPAL provided lower values of 70 µmol·l^−1^. The *k*_cat_ values ranged from 0.02 to 0.09 s^−1^. The best substrate of this group was PheAPAL with a relative catalytic efficiency of approximately 0.2 compared to the natural substrate AASAL (Table 2). TyrABAL oxidation resulted in low absorbance values, which excluded obtaining reliable kinetic parameters.

**Table 1:** Kinetic parameters of the oxidation of *N*-carboxyacyl aminoaldehydes by PsAMADH

Substrate	*K*_m_ (μmol·l^−1^)	*k*_cat_ (s^−1^)	*k*_cat*/*_*K*_*m*_ (mol^−1^·l·s^−1^)	*k*_cat*/*_*K*_*m*_ (relative)
APAL	136 ± 11	3.36 ± 0.20	24.7 × 10^3^	1.000
MalAPAL	597 ± 64	1.35 ± 0.10	2.3 × 10^3^	0.093
SucAPAL	513 ± 36	4.13 ± 0.20	8.1 × 10^3^	0.328
GltAPAL	506 ± 92	1.29 ± 0.14	2.5 × 10^3^	0.101
ABAL	103 ± 17	1.34 ± 0.07	13.0 × 10^3^	0.526
MalABAL	879 ± 83	1.28 ± 0.18	1.5 × 10^3^	0.061
SucABAL	138 ± 6	1.61 ± 0.07	11.7 × 10^3^	0.474
GltABAL	427 ± 33	1.49 ± 0.11	3.5 × 10^3^	0.142

The results were obtained using spectrophotometric measurements. *K*_m_ and *k*_cat_ values are presented as arithmetic means calculated by GraphPad Prism 8.0 program (nonlinear regression with excess substrate inhibition) using triplicated input data (see Fig. 3). APAL and ABAL were included as reference substrates

**Table 2:** Kinetic parameters of the oxidation of *N*-carboxylacyl and *N*-aminoacyl aminoaldehyde by PsALDH7 and ZmALDH7

Substrate	*K*_m_ (μmol·l^−1^]	*k*_cat_ (s^−1^)	*k*_cat*/*_*K*_*m*_ (mol^−1^·l·s^−1^)	*k*_cat*/*_*K*_*m*_ (relative)
*for PsALDH7*				
AASAL	172 ± 32	0.77 ± 0.09	4.5 × 10^3^	1.000
MalAPAL	2522 ± 203	0.19 ± 0.01	7.5 × 10^1^	0.017
MalABAL	703 ± 72	0.13 ± 0.01	1.8 × 10^2^	0.040
PheAPAL	74 ± 7	0.07 ± 0.00	9.5 × 10^2^	0.211
PheABAL	656 ± 55	0.04 ± 0.00	6.1 × 10^1^	0.014
TyrAPAL	252 ± 45	0.09 ± 0.01	3.6 × 10^2^	0.080
*for ZmALDH7*				
AASAL	191 ± 14	0.77 ± 0.04	4.0 × 10^3^	1.000
MalAPAL	1997 ± 142	0.15 ± 0.01	7.5 × 10^1^	0.019
MalABAL	1493 ± 225	0.18 ± 0.01	1.2 × 10^2^	0.030
PheAPAL	66 ± 12	0.06 ± 0.01	9.1 × 10^2^	0.228
PheABAL	457 ± 55	0.02 ± 0.00	4.4 × 10^1^	0.011
TyrAPAL	216 ± 56	0.06 ± 0.01	2.8 × 10^2^	0.070

The results were obtained using spectrophotometric measurements. *K*_m_ and *k*_cat_ values are presented as arithmetic means calculated by GraphPad Prism 8.0 program (nonlinear regression with excess substrate inhibition) using triplicated input data (see Fig. 3). AASAL was included as a reference substrate

**Table 3:** Kinetic parameters of the oxidation of *N*-aminoacyl aminoaldehydes by PsAMADH

Substrate	*K*_m_ (μmol·l^−1^)	*k*_cat_ (s^−1^)	*k*_cat*/*_*K*_*m*_ (mol^−1^·l·s^−1^)	*k*_cat*/*_*K*_*m*_ (relative)
APAL	129 ± 8	4.34 ± 0.17	33.6 × 10^3^	1.000
PheAPAL	818 ± 23	0.24 ± 0.00	2.9 × 10^2^	0.009
PheABAL	101 ± 10	0.17 ± 0.00	1.7 × 10^3^	0.051
TyrABAL	174 ± 42	0.22 ± 0.04	1.3 × 10^3^	0.039

The results were obtained using spectrophotometric measurements. *K*_m_ and *k*_cat_ values are presented as arithmetic means calculated by GraphPad Prism 8.0 program (nonlinear regression with excess substrate inhibition) using triplicated input data (see Fig. 3). APAL was included as a reference substrate

The *K*_m_ values calculated using a standard Michaelis–Menten nonlinear regression were as follows (in μmol·l^−1^): 36 (APAL), 159 (SucAPAL), 64 (ABAL) and 48 (SucABAL).

### Docking of the synthetic substrates into the active sites of ALDHs

**Fig. 4:**
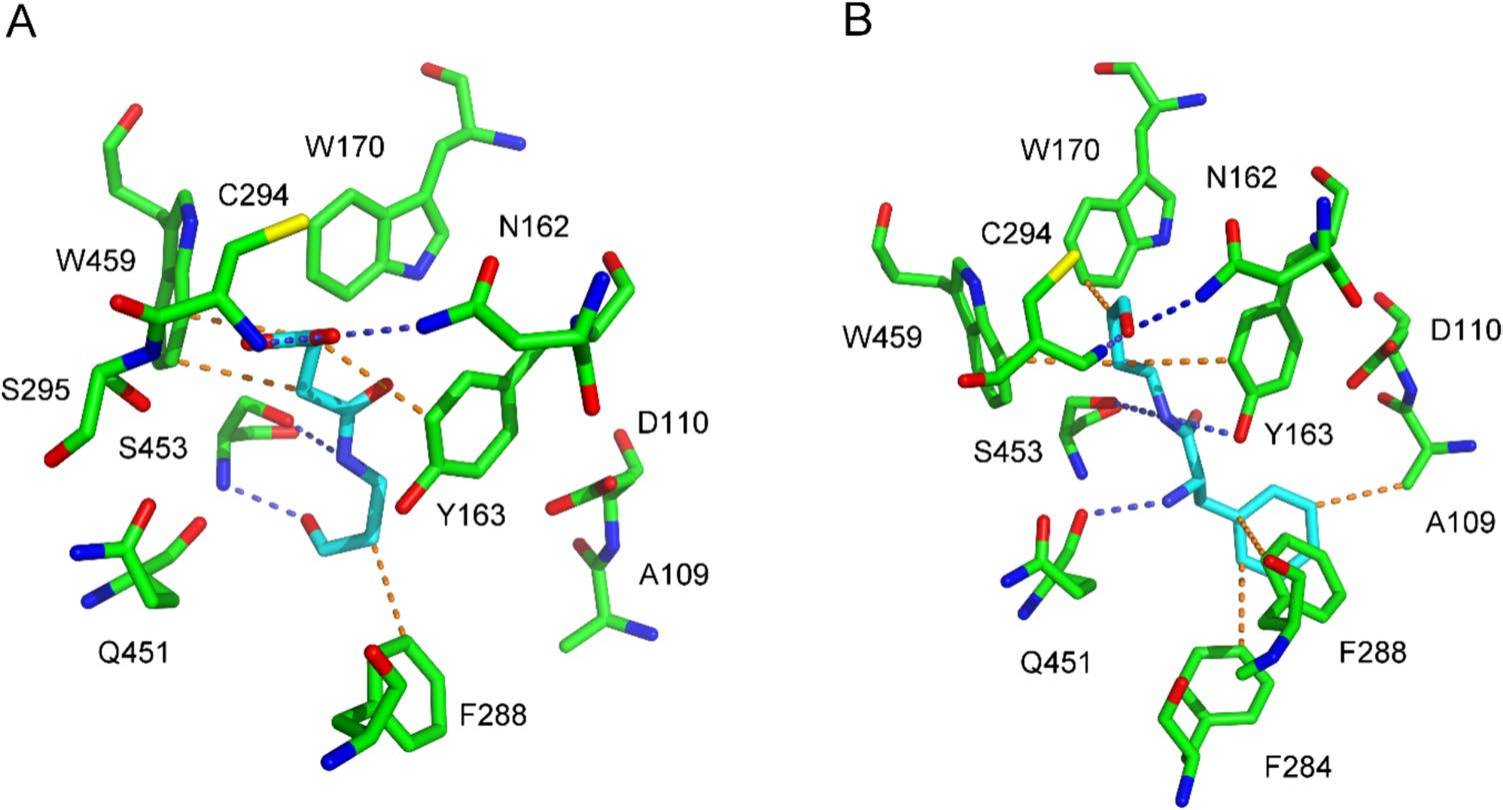
Molecular docking of SucAPAL and PheABAL into the active site of PsAMADH1. The enzyme–substrate complexes shown were obtained using the Achilles Blind Docking Server (https://bio-hpc.ucam.edu/achilles, accessed on 20 December 2023) and processed in PyMOL 1.3. **A** PsAMADH1–SucAPAL complex; **B** PsAMADH1–PheABAL complex. The active site residues are depicted in atom-coded colors, with carbons in green, nitrogens in blue, oxygens in red, and sulfur in yellow. The modeled hydrophobic interactions of the substrates are represented by orange dashes, while hydrogen bonds are indicated by blue dashes, with carbons of the substrates shown in cyan

The binding of the synthesized compounds at the active site of PsAMADH1, PsAMADH2, and ZmALDH7 was modeled using the Achilles Blind Docking Server (Sánchez-Linares et al. 2012). MalAPAL, SucAPAL, GltAPAL, MalABAL, SucABAL, and GltABAL were all docked in the substrate channel of PsAMADH1 with their aldehyde group pointing away from the catalytic Cys294. The corresponding binding energies ranged between -6.20 and -5.40 kcal·mol^−1^. In the obtained complexes, MalAPAL and MalABAL interact with Tyr163, Leu166, and Trp459 through hydrophobic interactions, and also with Ser453 via the amide nitrogen of the substrate, forming a hydrogen bridge. The carboxylic group of the substrate forms hydrogen bridges with Asn162, Cys294, and Ser295. SucAPAL shows additional hydrophobic interactions with Phe288. SucABAL also interacts with Trp170, and similarly to SucAPAL it does interact with Leu166 (Fig. 4). Finally, GltAPAL and GltABAL interact with Tyr163, Trp170, Phe288, and Trp459 (hydrophobic interactions). Asn162, Tyr163, Cys294, Ser295, and Ser453 participate in hydrogen bonds. Gln451 is involved in hydrogen bonding with SucABAL and GltABAL.

The docked pose of PheABAL in the active site of PsAMADH1, with a calculated binding energy of  -6.70 kcal·mol^−1^, is characterized by the aldehyde group being oriented towards the catalytic cysteine. The substrate's phenyl group is engaged in hydrophobic interactions with Ala109, Phe284, Phe288, while the aliphatic chain interacts with Tyr163, Trp170, and Trp459 (Fig. 4). The primary amino group of PheABAL shows a bonding interaction with the backbone oxygen of Gln451 whereas the amide hydrogen of PheABAL makes a hydrogen bridge to the side-chain oxygens of Tyr163 and Ser453. The aldehyde oxygen of the substrate participates in a hydrogen bond with the side-chain amide of Asn162. Similarly, Asn162, Tyr163, Trp170, and Trp459 are involved in binding of TyrABAL, with a calculated binding energy of -6.20 kcal·mol^−1^. PheAPAL appears in a pose in which its aldehyde group is positioned away from the catalytic Cys294. Unfortunately, TyrAPAL could not be docked at the active site of PsAMADH1. Similarly, none of the studied carboxylate-containing compounds could be docked at the active site of PsAMADH2 in a typical substrate binding interaction involving Tyr163 and Trp288. Instead, their binding pose in this isozyme is located near Cys294 and Phe395, with calculated binding energies around  -5 kcal·mol^−1^ for the *N*-carboxyacyl compounds and around -6 kcal·mol^−1^ for the *N*-aminoacyl derivatives.

**Fig. 5:**
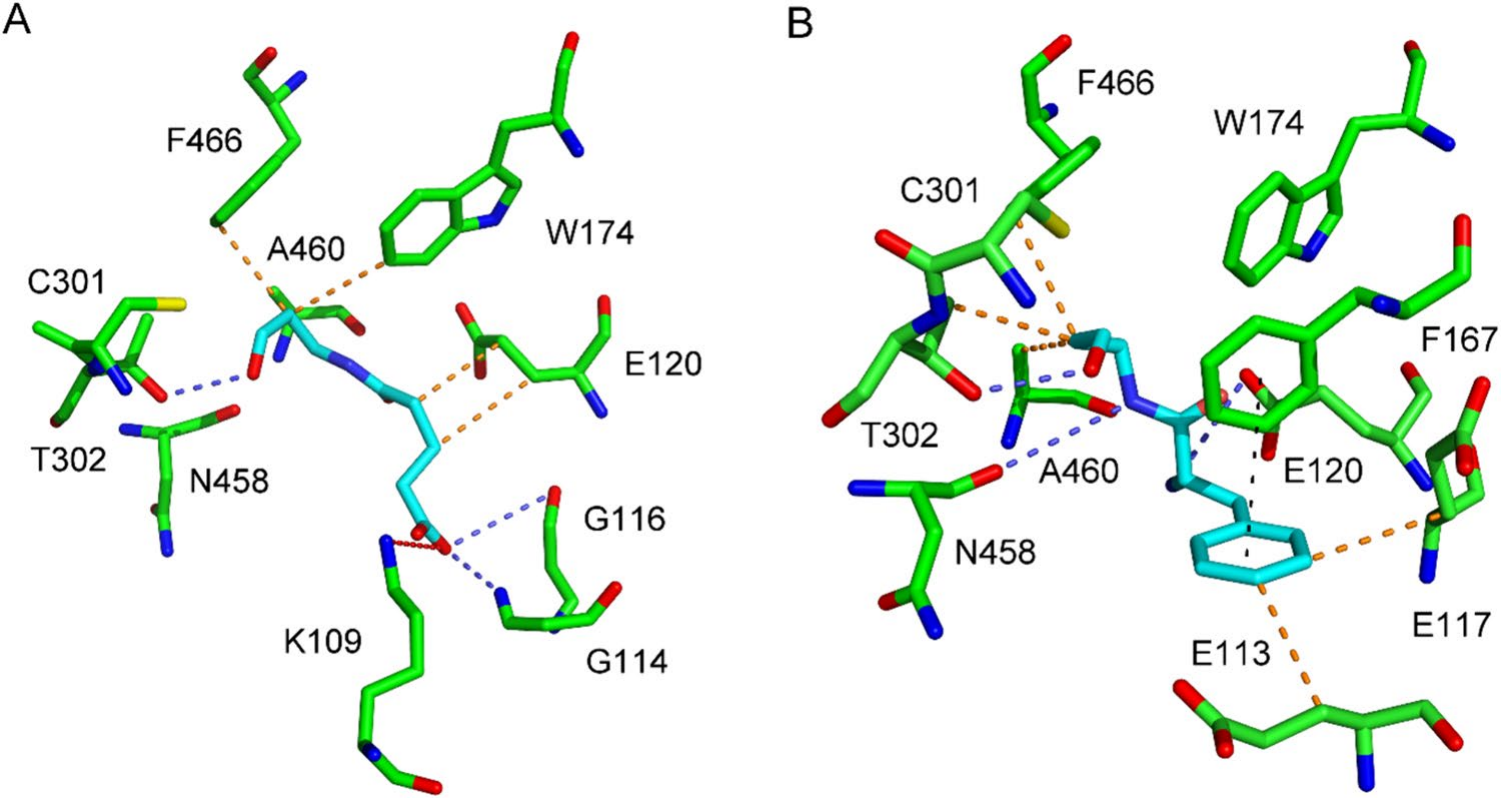
Molecular docking of GltAPAL and PheAPAL into the the active site of ZmALDH7. The enzyme–substrate complexes shown were obtained using the Achilles Blind Docking Server (https://bio-hpc.ucam.edu/achilles, accessed on 20 December 2023) and processed in PyMOL 1.3. **A** ZmALDH7–GltAPAL complex; **B** ZmALDH7–PheAPAL complex. The active site residues are depicted in atom-coded colors, with carbons in green, nitrogens in blue, oxygens in red, and sulfur in yellow. The modeled hydrophobic interactions of the substrates are represented by orange dashes, while hydrogen bonds are indicated by blue dashes, with carbons of the substrates shown in cyan. The pi-stacking interaction is shown as a black dashed line

The docked poses of MalAPAL and MalABAL in the active site of ZmALDH7 are characterized by the orientation of the carbonyl group towards the catalytic Cys301. Hydrophobic interactions involve Glu113, Glu120, and Phe167 or Trp174. Hydrogen bonds are formed via the substrate's amide nitrogen or carboxylic group with Gly116 and Thr296, respectively. Similarly, docked SucAPAL and SucABAL show hydrophobic interactions with Phe167 and Trp174, hydrogen bonds involve Gly114 and Thr302 or additionally Ala460 (SucABAL). Interestingly, docking of SucAPAL provided an inverted position compared to SucABAL with its aldehyde group pointing to Glu113 (not shown). GltAPAL makes hydrophobic interactions with Glu120, Trp174, Ala460, and Phe466 (Fig. 5). The carboxylic group of the substrate interacts with Lys109 (an ion pair) and Gly114 + Gly116 (hydrogen bonds). Another hydrogen bond connects the carbonyl group and Thr302. The GltABAL chain interacts hydrophobically with Glu113, Phe167, and Trp174. Hydrogen bonds are shared with Glu120 and Thr296. The binding energies for the carboxylate containing substrates ranged between -5.40 and -4.50 kcal·mol^−1^. The molecule of PheAPAL was docked in ZmALDH7, positioning its aldehyde group near the catalytic Cys301 (binding energy of -6.20 kcal·mol^−1^). Its primary amino group forms a hydrogen bond interaction with Glu120, and the amide nitrogen interacts similarly with Asn458 (Fig. 5). Potential hydrophobic interactions involve Glu113, Glu117, Phe167, Thr302, Ala460, and Phe466. Additionally, Phe167 interacts through a stacking interaction with the substrate ring. The docking server provided a similar pose also for PheABAL (-6.30 kcal·mol^−1^). The hydroxyl group of the docked TyrABAL (-6.30 kcal·mol^−1^) forms additional interactions via hydrogen bonds with Lys109, Thr296, and Arg300. Unfortunately, TyrAPAL could not be successfully docked at the active site of ZmALADH7.

## Discussion

ALDHs from the families 9 and 10 are known as aminoaldehyde dehydrogenases (AMADHs) due to their efficient activity with the aminoaldehydes APAL and ABAL produced by polyamine oxidation. APAL is recognized as a reactive compound that needs neutralization in the cell (Šebela and Rašková 2023). The peroxisomal localization of many plant AMADHs such as those from Arabidopsis (Reumann et al. 2009; Jacques et al. 2020), or rice (Mitsuya et al. 2009), correlates well with the production of APAL by peroxisomal polyamine oxidases (Samanta et al. 2023; Salvi and Tavladoraki 2020). In addition to this detoxifying role, the AMADH reaction produces compatible osmolytes (γ-aminobutyric acid, glycine betaine) or their precursors (β-alanine) and contributes to lysine metabolism by the oxidation of trimethylaminobutyraldehyde in carnitine biosynthesis (Kopečný et al. 2013; Jacques et al. 2020). ALDH7 enzymes have been shown to contribute to lysine catabolism by converting AASAL to α-aminoadipic acid and protect against stress by producing compatible osmolytes and oxidizing toxic aldehydes (Brocker et al. 2010; Končitíková et al. 2015). ALDH7, 9 and 10 share several common substrates. Both plant and human ALDH7s oxidize the aminoaldehydes that are known to be efficient substrates for ALDH9 and ALDH10, such as APAL, ABAL, 4-guanidinobutyraldehyde (GBAL), and betaine aldehyde (BAL). Apart from BAL, which provides *K*_m_ values of 40–120 μmol·l^−1^, the other compounds show much higher millimolar *K*_m_ values, indicating less favorable binding at the active site (Brocker et al. 2010; Končitíková et al. 2015). With the exception of GBAL, which provides *k*_cat_ values at a comparable level to those for AASAL, other aminoaldehydes show lower oxidation rates characterized by *k*_cat_ values of 10^–2^–10^–1^ s^−1^ (Brocker et al. 2010; Končitíková et al. 2015). On the other hand, AASAL, one the best substrates of ALDH7s, is only negligibly oxidized by the broadly specific ALDH10 isoenzyme 1 from tomato with a rate of < 1% compared to that achieved with APAL at 1 mmol·l^−1^ concentrations (our unpublished results). To our knowledge, no common substrate with a comparable binding and turnover rate is available for the enzymes of the ALDH families 7, 9 and 10, which would for example allow kinetic measurements of the total production rate of compatible osmolytes in tissue extracts. BAL would be a good candidate but it is not oxidized efficiently by all isoenzymes of plant ALDH10s. We conducted a screening study with *N*-carboxyacyl aminoaldehydes and Phe/Tyr derivatives of APAL and ABAL to look for a synthetic candidate.

Synthesis of the studied *N*-carboxyacyl derivatives was inspired by the previously discovered efficient substrate properties of *N*-adipoyl-APAL towards the ALDH10 isoenzymes from pea (Frömmel et al. 2015), or γ-glutamylated APAL and ABAL towards the PauC ALDH enzyme from *Pseudomonas aeruginosa* (Cardona-Cardona et al. 2022). PheAPAL, PheABAL, TyrAPAL, and TyrABAL were included as *N*-α-aminoacylated compounds with an aromatic ring opposite the aldehyde group expected to provide hydrophobic and stacking interactions at the active site. The best known substrates of the studied ALDH enzymes (typically natural compounds) show *K*_m_ values of 10^–6^–10^–5^ mol·l^−1^ and *k*_cat_ values of 1–10 s^−1^ (Tylichová et al. 2010; Končitíková et al. 2015). All new compounds were substrates of PsAMADH, PsALDH7 and ZmALDH7 as demonstrated by mass spectrometry of the reaction mixtures and spectrophotometric activity assays. The measured *k*_cat_ values were relatively high for PsAMADH and the *N*-carboxyacyl derivatives (1–4 s^−1^) and appeared at the 10^–1^ s^−1^ level for PsAMADH and the *N*-phenylalanyl/tyrosyl derivatives as well as for both ALDH7s and MalAPAL/MalABAL. On the other hand, the reactions of *N*-phenylalanyl/tyrosyl derivatives with the ALDH7s were characterized by lower *k*_cat_ values of 10^–2^ s^−1^. Except for a very few compounds, the *K*_m_ values determined with PsAMADH, PsALDH7 and ZmALDH7 were around 500 μmol·l^−1^ or even more up to 2.5 mmol·l^−1^. This indicates that the binding in the active sites is typically less favorable for the synthetic compounds than in the case of the natural substrates and the oxidizing activity is either comparable or goes down by one or two orders of magnitude. The exceptions were SucABAL and PheABAL for PsAMADH and PheAPAL for the two ALDH7s, which provided the lowest *K*_m_ values comparable to the best natural substrates of the enzymes. Molecular docking calculations were thus performed to elucidate this observation.

The active site of plant ALDH10s is accessible via the substrate channel, which contains many conserved residues, but also shows differences that influence substrate specificity. The amino acid residues within this cavity, which are involved in the process of substrate binding, were studied either through site-directed mutagenesis (Kopečný et al. 2011) or by comparing available crystal structures of different ALDH10s and correlating them with the respective kinetic properties (Kopečný et al. 2013). Protonated ω-aminoaldehydes are cationic and thus readily recognized by the negatively charged and highly conserved aspartate residues at the channel entrance (Asp110 and 113 in PsAMADH1 and 2). Aromatic residues, namely Tyr163, Trp170, Trp288, and Trp459, are responsible for the high affinity of PsAMADH2 to APAL and ABAL (Kopečný et al. 2011, 2013). Trp288 is replaced by Phe or Ala in particular ALDH10s (e.g. PsAMADH1 and tomato ALDH10 isoenzyme 1, respectively), which decreases the affinity for APAL but facilitates the binding of another natural substrate trimethylaminobutyraldehyde. The highly conserved Tyr163 and Trp170, located in the middle section of the substrate cavity, are crucial for anchoring the substrate chain. Tyr163 forms a hydrogen bond with the substrate amino group (Kopečný et al. 2013). A previous study with different *N*-acyl aliphatic aminoaldehyde substrates of plant ALDH10s indicated the importance of Tyr163 for substrate binding by the formation of a hydrogen bond involving its hydroxyl and substrate amido group (Frömmel et al. 2015).

The procedure of isolating native PsAMADH used leads to preparations with a prevailing representation of PsAMADH1 isoenzyme (Šebela et al. 2000) and the present kinetic parameters *K*_m_ and *k*_cat_ determined with APAL and ABAL correspond to the data for this isoenzyme (Tylichová et al. 2010). For PsAMADH1 structure, the *N*-carboxyacyl derivatives exhibited docked poses with multiple hydrophobic and hydrogen bonds. Despite the fact that the docked compounds have their aldehyde group oriented away from the catalytic cysteine (Fig. 4), they establish the corresponding amino acid bonding contacts in the active site as the natural aminoaldehyde substrates. This orientation implies that higher substrate concentrations are required to enhance the likelihood of proper binding in a productive mode, facilitating enzymatic conversion. This could explain the observed increase in the *K*_m_ values compared to APAL or ABAL. The reason for SucABAL showing the lowest *K*_m_ value among the group of *N*-carboxyacyl derivatives is not entirely clear from the docking experiments. However, it appears that additional hydrophobic bonds, such as that with Trp170, are involved compared to the binding of SucAPAL and the other substrates in this group. The flexibility of the butyraldehyde moiety in SucABAL may also play a positive role in this context. PheABAL is anchored in the active site by six hydrophobic bonds and two of them involve the aromatic ring of the substrate. This ring involvement probably contributes to the low *K*_m_ value of 100 μmol·l^−1^ observed. Also PheAPAL forms several hydrophobic interactions involving its aromatic ring but the aldehyde group is not oriented properly to allow the attack of the catalytic cysteine thiol group.

The active site of plant ALDH7s has been well explored by solving the crystal structure of ZmALDH7 (Končitíková et al. 2015). The substrate-binding pocket, which is funnel-shaped, is surrounded by the hydrophobic residues Phe167, Ala170, Trp174 and Phe466. Its entrance part is guarded by Glu120 and Arg300. The former residue was first reported to be important for the substrate specificity of seabream (*Acanthopagrus schlegelii*) ALDH7, as it binds the amino group of the natural substrate AASAL. Arg300 maintains the overall conformation of the active site for the nucleophilic attack of the catalytic Cys301 on the substrate aldehyde carbonyl group (Tang et al. 2008). Human ALDH7 was co-crystallized with aminoadipate, which is the reaction product of AASAL substrate conversion (Luo and Tanner 2015). Hydrogen bonds anchor the bound product to Gly461 and Ala462 (the human ALDH7A1 numbering). Another hydrogen bond involves Thr303. Ion pairs are formed with Glu121 and Arg301. The aliphatic chain of the aminoadipate interacts with Phe168, Trp175, and Phe468. As suggested by docking results, these hydrophobic residues would interact with the aliphatic chain of the efficient nonanal substrate as well (Brocker et al. 2010). The high millimolar *K*_m_ values of PsALDH7 for the aminoaldehydes GBAL and ABAL indicate that the presence of the terminal carboxyl group in AASAL and its interaction with Arg300 could be important for high-affinity substrate binding (Končitíková et al. 2015). Consistent with previous observations, molecular docking analyses within this study confirm that Phe167, Trp174, Ala460 and Phe466 bind the carbon chain of the new substrates. PheAPAL, which shows the lowest *K*_m_ value towards PsALDH7 and ZmALDH7, is additionally bound by its amino group with Glu120 (hydrogen bridge) and provides many other hydrophobic and hydrogen bonding interactions, and also pi-pi stacking of the substrate ring with the Phe167 ring, which facilitate its recognition as a good substrate (Fig. 5). Such a stacking interaction or ion-pair/hydrogen bond interaction with Glu120 is missing for the *N*-carboxyacyl derivatives, which showed high *K*_m_ values of 10^–3^ mol·l^−1^.

In conclusion, the original idea to design aminoaldehydes terminated by a carboxylic group or an aromatic ring opposite the aldehyde group as shared substrates of ALDH10s and ALDH7s resulted in 10 compounds. All of them were substrates of the enzymes. Although no compound exhibited the same level of substrate properties for both ALDH families, we show that these enzymes may possess more common substrates than expected. The measured kinetic parameters correlated with the docking and could somehow elucidate the observed kinetic differences. The presence of the aromatic ring and the primary amino group in PheAPAL/PheABAL allow interactions with Phe167 (multiple bonds) and Glu120, respectively, which obviously contributes to the proper binding in the active site of ALDH7s, particularly in the case of PheAPAL. Similarly, hydrophobic interactions of the substrate ring involving Phe284 or Phe288 facilitate binding of PheABAL in PsAMADH1 compared to PheAPAL. On the other hand, the terminal carboxylate group of *N*-malonyl-, *N*-succinyl- and *N*-glutaryl-derivatives can form hydrogen bonds with Asn162, Cys294 and Ser295 in PsAMADH1. However, this substrate binding is not productive for the catalytic attack of Cys294 at the aldehyde group. Higher concentrations are necessary for the proper binding, which increases the *K*_m_ value. In ALDH7s, the carboxylate of the substrates is attracted by Lys109 and thus the aldehyde group is oriented properly.

## Supplementary Information

Below is the link to the electronic supplementary material.

Supplementary file1 (DOCX 2126 KB)

Supplementary file2 (DOCX 308 KB)

## Data Availability

The data that support the findings of this study are available from the corresponding author, MŠ, upon reasonable request.

## Abbreviations

AASAL: L-α-Aminoadipate-δ-semialdehyde
ABAL: 4-Aminobutyraldehyde
ALDH: Aldehyde dehydrogenase
AMADH: Aminoaldehyde dehydrogenase
APAL: 3-Aminopropionaldehyde
BAL: Betaine aldehyde
Boc-Phe-OH: *N*-(*tert*-Butoxycarbonyl)-l-phenylalanine
Boc-Tyr(*t*Bu)-OH: *N*-(*tert*-Butoxycarbonyl)-O-*tert*-butyl-l-tyrosine
CHCA: α-Cyano-4-hydroxycinnamic acid
CTAB: Cetyltrimethylammonium bromide
DIPEA: *N*,*N*-Diisopropylethylamine
GBAL: 4-Guanidinobutyraldehyde
GltABAL: 4-[(Glutaryl)amino]butyraldehyde i.e. 5-oxo-5-[(4-oxobutyl)amino]pentanoic acid
GltAPAL: 3-[(Glutaryl)amino]propionaldehyde i.e. 5-oxo-5-[(3-oxopropyl)amino]pentanoic acid
MalABAL: 4-[(Malonyl)amino]butyraldehyde i.e. 3-oxo-3-[(4-oxobutyl)amino]propanoic acid
MalAPAL: 3-[(Malonyl)amino]propionaldehyde i.e. 3-oxo-3-[(3-oxopropyl)amino]propanoic acid
PheABAL: 4-[(L-phenylalanyl)amino]butyraldehyde i.e. 2-amino-*N*-(4-oxobutyl)-3-phenylpropanamide
PheAPAL: 3-[(L-phenylalanyl)amino]propionaldehyde i.e. 2-amino-*N*-(3-oxopropyl)-3-phenylpropanamide
PsALDH7: Aldehyde dehydrogenase 7 from *Pisum sativum*
PsAMADH: Aminoaldehyde dehydrogenase from *Pisum sativum*
SucABAL: 4-[(Succinyl)amino]butyraldehyde i.e. 4-oxo-4-[(4-oxobutyl)amino]butanoic acid
SucAPAL: 3-[(Succinyl)amino]propionaldehyde i.e. 4-oxo-4-[(3-oxopropyl)amino]butanoic acid
TyrABAL: 4-[(L-tyrosyl)amino]butyraldehyde i.e. 2-amino-3-(4-hydroxyphenyl)-*N*-(4-oxobutyl)propanamide
TyrAPAL: 3-[(L-tyrosyl)amino]propionaldehyde i.e. 2-amino-3-(4-hydroxyphenyl)-*N*-(3-oxopropyl)propanamide
ZmALDH7: Aldehyde dehydrogenase 7 from *Zea mays*
