# Supplementary material for: N-carboxyacyl and N-α-aminoacyl derivatives of aminoaldehydes as shared substrates of plant aldehyde dehydrogenases 10 and 7
Source: Amino Acids. 2024 Aug 29;56(1):52. doi: 10.1007/s00726-024-03415-4 (PMC11362210; doi:10.1007/s00726-024-03415-4)
Supplement: Supplementary file 1 — Supplementary file1 (DOCX 2126 KB) [file 726_2024_3415_MOESM1_ESM.docx]

***N*-carboxyacyl and *N*-α-aminoacyl derivatives of aminoaldehydes as shared substrates of plant aldehyde dehydrogenases 10 and 7**

**Michaela Masopustová^1^, Adam Goga^1^, Miroslav Soural^2^, Martina Kopečná^3^, and Marek Šebela^1^**

^1^Department of Biochemistry, ^2^Department of Organic Chemistry, and Department of Experimental Biology^3^, Faculty of Science, Palacký University, Olomouc, Czech Republic

**SUPPLEMENTARY FILE 1**

**Purity check of PsALDH7, ZmALDH7 and native PsAMADH**

SDS-PAGE (Laemmli buffer system) resolving gels (12%) show the purity of the three enzymes used in this study. **Left panel**: protein marker with the indicated molecular mass values, PsALDH7 (8 μg), ZmALDH7 (8 μg). **Right panel**: protein marker with the indicated molecular mass values, PsAMADH after ion exchange chromatography on a MonoQ column (56 μg), PsAMADH after affinity chromatography on 5´-AMP- Sepharose (1.5 μg). Arrows indicate the isoenzymes PsAMADH1 (top) and PsAMADH2 (bottom). The gels were stained by Coomassie Brilliant Blue G-250 following a colloidal staining protocol. The identities of the purified proteins were verified using nanoLC-MALDI-TOF MS/MS of their tryptic in-gel digests.

Results of the identifications were as follows:

- PsAMADH1 (gi|291047670, score: 1053, sequence coverage: 31 %, 15 identified peptides),

- PsAMADH2 (gi|291047674, score: 640, sequence coverage: 26 %, 10 identified peptides),

- ZmALDH7 (gi|770386322, score: 621, sequence coverage: 59 %, 19 identified peptides),

- PsALDH7 (gi|118514, score: 1204, sequence coverage: 43 %, 19 identified peptides).


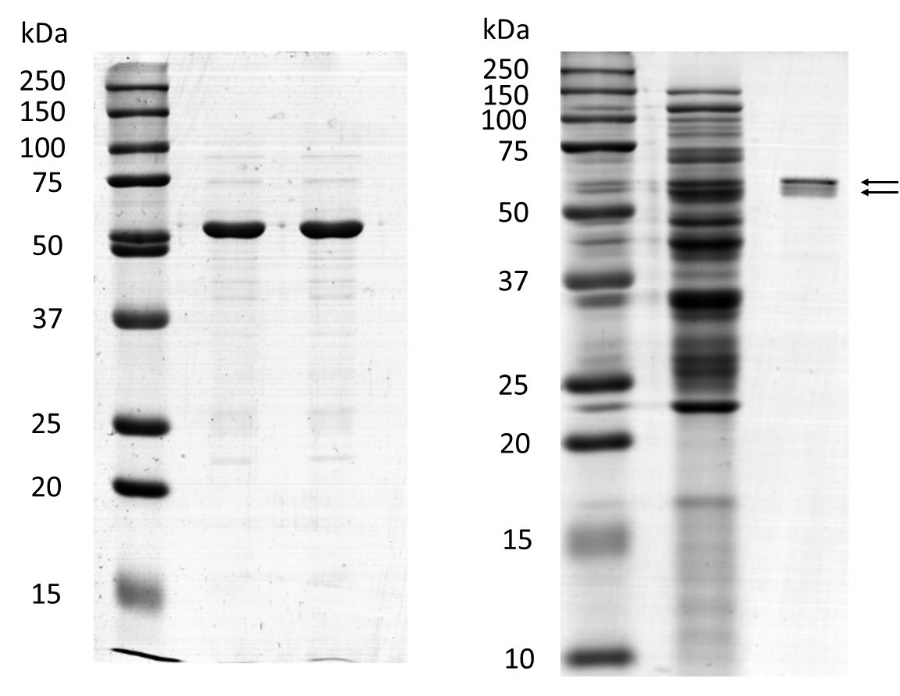


**A multiple amino acid alignment o ZmALDH7, PsALDH7, PsAMADH1 and PsAMADH2 obtained using Clustal W**

The yellow shadowing highlights important residues of the active sites. Asterisks indicate Asn162, Glu260, and Cys294 important for the catalytic mechanism of PsAMADHs (Tylichová M et al., J. Mol. Biol. 396, 870-882, 2010), as well as Glu120, Arg300, and Cys301, which are crucial for the conversion of aminoadipic semialdehyde by plant ALDH7s (Končitíková et al., Biochem. J. 468, 109-123, 2015). For the 3-D architecture of the active sites and molecular docking of the novel substrates, see Figs. 4 and 5 in the article. The accession codes refer to the UniProt database.

**MalAPAL with protecting groups: ethyl 3-[(3,3-diethoxypropyl)amino]-3-oxopropanoate**

^1^H NMR (500 MHz, CHLOROFORM-*d*) δ ppm 1.18 - 1.23 (m, 6 H) 1.27 (t, *J*=7.16 Hz, 4 H) 1.81 - 1.87 (m, 2 H) 3.26 (s, 2 H) 3.35 - 3.41 (m, 2 H) 3.50 (dq, *J*=9.38, 6.99 Hz, 2 H) 3.66 (dq, *J*=9.45, 7.06 Hz, 2 H) 4.15 - 4.21 (m, 2 H) 4.55 (t, *J*=5.30 Hz, 1 H) 7.28 - 7.35 (m, 1 H). ^13^C NMR (125 MHz, CHLOROFORM-*d*) δ ppm 14.03, 15.25, 32.97, 35.62, 41.45, 61.39, 61.81, 102.03, 164.79, 169.25.

Note: Singlet at 5.25 ppm belongs to CH_2_Cl_2_ (residual solvent in the sample)

Note: Signal at 53.38 ppm belongs to CH_2_Cl_2_ (residual solvent in the sample)

**SucAPAL with protecting groups: ethyl 4-[(3,3-diethoxypropyl)amino]-4-oxobutanoate**

^1^H NMR (500 MHz, CHLOROFORM-*d*) δ ppm 1.20 - 1.28 (m, 9 H) 1.79 - 1.85 (m, 2 H) 2.42 - 2.46 (m, 2 H) 2.65 (t, *J*=7.02 Hz, 2 H) 3.33 - 3.39 (m, 2 H) 3.51 (dq, *J*=9.38, 6.99 Hz, 2 H) 3.68 (dq, *J*=9.45, 7.06 Hz, 2 H) 4.14 (q, *J*=7.16 Hz, 2 H) 4.56 (t, *J*=5.16 Hz, 1 H) 6.14 - 6.23 (m, 1 H). ^13^C NMR (125 MHz, CHLOROFORM-*d*) δ ppm 14.14, 15.31, 29.59, 31.10, 32.91, 35.58, 60.58, 61.91, 102.41, 171.04, 172.93.

Note: Singlet at 5.25 ppm belongs to CH_2_Cl_2_ (residual solvent in the sample)

Note: Signal at 53.38 ppm belongs to CH_2_Cl_2_ (residual solvent in the sample)

**GltAPAL with protecting groups: ethyl 5-[(3,3-diethoxypropyl)amino]-5-oxopentanoate**

^1^H NMR (500 MHz, CHLOROFORM-*d*) δ ppm 1.20 - 1.29 (m, 9 H) 1.80 - 1.85 (m, 2 H) 1.92 - 2.00 (m, 2 H) 2.18 - 2.24 (m, 2 H) 2.34 - 2.39 (m, 2 H) 3.34 - 3.40 (m, 2 H) 3.51 (dq, *J*=9.34, 7.10 Hz, 2 H) 3.68 (dq, *J*=9.34, 7.10 Hz, 2 H) 4.10 - 4.17 (m, 4 H) 4.57 (t, *J*=5.16 Hz, 1 H) 6.09 - 6.17 (m, 1 H). ^13^C NMR (125 MHz, CHLOROFORM-*d*) δ ppm 14.19, 15.31, 20.89, 32.92, 33.41, 35.44, 35.64, 60.31, 61.97, 102.53, 171.80, 173.13.

Note: Singlet at 5.25 ppm belongs to CH_2_Cl_2_ (residual solvent in the sample)

**MalABAL with protecting groups: ethyl 3-[(4,4-diethoxybutyl)amino]-3-oxopropanoate**

^1^H NMR (400 MHz, CHLOROFORM-*d*) δ ppm 1.17 (t, *J*=7.10 Hz, 6 H) 1.22 - 1.28 (m, 3 H) 1.54 - 1.66 (m, 4 H) 3.23 - 3.31 (m, 4 H) 3.46 (dq, *J*=9.39, 7.02 Hz, 2 H) 3.61 (dq, *J*=9.39, 7.10 Hz, 2 H) 4.16 (q, *J*=7.17 Hz, 2 H) 4.45 (t, *J*=5.27 Hz, 1 H) 7.07 - 7.19 (m, 1 H). ^13^C NMR (125 MHz, CHLOROFORM-*d*) δ ppm 13.99, 15.27, 24.53, 30.95, 39.27, 41.17, 61.29, 61.47, 102.55, 164.87, 169.57.

**SucABAL with protecting groups: ethyl 4-[(4,4-diethoxybutyl)amino]-4-oxobutanoate**

^1^H NMR (400 MHz, CHLOROFORM-*d*) δ ppm 1.13 - 1.18 (m, 6 H) 1.19 - 1.24 (m, 3 H) 1.49 - 1.65 (m, 4 H) 2.38 - 2.44 (m, 2 H) 2.57 - 2.64 (m, 2 H) 3.17 - 3.26 (m, 2 H) 3.45 (dq, *J*=9.47, 6.99 Hz, 2 H) 3.60 (dq, *J*=9.39, 7.02 Hz, 2 H) 4.10 (q, *J*=7.10 Hz, 2 H) 4.44 (t, *J*=5.27 Hz, 1 H) 5.86 - 5.97 (m, 1 H). ^13^C NMR (125 MHz, CHLOROFORM-*d*) δ ppm 14.22, 15.39, 24.66, 29.74, 31.08, 31.18, 39.40, 60.71, 61.46, 102.72, 171.41, 173.11.

**GltABAL with protecting groups: ethyl 5-[(4,4-diethoxybutyl)amino]-5-oxopentanoate**

^1^H NMR (500 MHz, CHLOROFORM-*d*) δ ppm 1.17 (t, *J*=7.02 Hz, 6 H) 1.23 (t, *J*=7.16 Hz, 3 H) 1.52 - 1.65 (m, 4 H) 1.89 - 1.95 (m, 2 H) 2.19 (t, *J*=7.45 Hz, 2 H) 2.29 - 2.36 (m, 2 H) 3.19 - 3.28 (m, 2 H) 3.46 (dq, *J*=9.34, 7.10 Hz, 2 H) 3.62 (dq, *J*=9.45, 7.06 Hz, 2 H) 4.05 - 4.14 (m, 2 H) 4.45 (t, *J*=5.30 Hz, 1 H) 5.77 (br. s., 1 H). ^13^C NMR (125 MHz, CHLOROFORM-*d*) δ ppm 14.19, 15.29, 20.96, 24.57, 31.02, 33.35, 35.56, 39.21, 60.35, 61.42, 102.63, 172.05, 173.23.

**PheAPAL with protecting groups: Boc-PheAPAL diethylacetal**

^1^H NMR (500 MHz, CHLOROFORM-*d*) δ ppm 1.16 (t, *J*=7.02 Hz, 6 H) 1.36 - 1.42 (m, 9 H) 1.63 - 1.76 (m, 2 H) 3.04 (d, *J*=6.01 Hz, 2 H) 3.29 (d, *J*=6.01 Hz, 2 H) 3.37 - 3.46 (m, 2 H) 3.55 - 3.64 (m, 2 H) 4.18 - 4.33 (m, 1 H) 4.43 (t, *J*=5.01 Hz, 1 H) 5.02 (br. s, 1 H) 6.32 (br. s, 1 H) 7.17 - 7.24 (m, 3 H) 7.26 - 7.31 (m, 2 H). ^13^C NMR (125 MHz) δ ppm 15.3, 28.2, 32.7 34.1, 35.4, 38.9, 61.7, 69.9, 100.1, 126.8, 128.6, 129.2, 136.8, 17.7.

**PheABAL with protecting groups: Boc-PheABAL diethylacetal**

^1^H NMR (500 MHz, CHLOROFORM-*d*) δ ppm 1.19 (t, *J*=7.16 Hz, 6 H) 1.41 (s, 9 H) 1.43-1.55 (m, 4 H) 2.98 - 3.11 (m, 2 H) 3.18 (q, *J*=6.30 Hz, 2 H) 3.46 (ddd, *J*=9.31, 7.02, 2.29 Hz, 2 H) 3.61 (dd, *J*=9.31, 7.02 Hz, 2 H) 4.20 - 4.34 (m, 1 H) 4.42 (t, 1 H, *J*=5.10) 4.99 - 5.16 (bs, 1 H) 5.79 - 5.96 (bs, 1 H) 7.18 - 7.25 (m, 3 H) 7.27 - 7.31 (m, 2 H). ^13^C NMR (125 MHz) δ ppm 15.3, 24.5, 25.6, 28.3, 30.8, 33.9, 38.8, 39.1, 61.3, 102.5, 126.9, 128.6, 129.3, 136.8, 171.0.

**TyrAPAL with protecting groups: Boc-Tyr(tBu)-APAL diethylacetal**

^1^H NMR (500 MHz, CHLOROFORM-*d*) δ ppm 1.14 (t, *J*=7.02 Hz, 6 H) 1.29 (s, 9 H) 1.36 (s, 9 H) 1.62 - 1.74 (m, 2 H) 2.90 - 3.00 (m, 2 H) 3.24 (dd, *J*=6.30, 5.40 Hz, 2 H) 3.36 - 3.45 (m, 2 H) 3.53 - 3.62 (m, 2 H) 4.11 - 4.29 (m, 1 H) 4.42 (t, *J*=5.20 Hz, 1 H) 5.11 (br. s, 1 H) 6.47 (br. s, 1 H) 6.87 (d, *J*=8.60 Hz, 2 H) 7.05 (d, *J*=8.30 Hz, 2 H). ^13^C NMR (125 MHz, CHLOROFORM-*d*) δ ppm 15.2, 28.2, 28.7, 32.7, 35.3, 38.1, 61.6, 61.8, 78.2, 102.0, 124.1, 129.6, 131.5, 154.2, 170.9.

Note: Signals at 1.2 ppm and 3.7 ppm belong to residual ethanol

Note: Signals at 18.2 ppm and 58.1 ppm belong to residual ethanol

**TyrABAL with protecting groups: Boc-Tyr(tBu)-ABAL diethylacetal**

^1^H NMR (500 MHz, DMSO-*d*_6_) δ ppm 1.06 (t, *J*=7.02 Hz, 6 H) 1.22 (s, 9 H) 1.26 (s, 9 H) 1.35 (d, *J*=7.16 Hz, 2 H) 1.44 (dd, *J*=8.02, 5.73 Hz, 2 H) 2.96 - 3.06 (m, 2 H) 3.34 - 3.42 (m, 2 H) 3.46 - 3.55 (m, 2 H) 4.01 - 4.09 (m, 1 H) 4.40 (t, *J*=5.58 Hz, 1 H) 6.81 (d, *J*=8.31 Hz, 2 H) 7.09 (d, *J*=8.31 Hz, 2 H) 7.76 (tt, *J*=5.73, 1.00 Hz, 1 H). ^13^C NMR (125 MHz, DMSO-*d*_6_) δ ppm 15.9, 25.0, 28.7, 29.0, 29.1, 31.2, 38.9, 56.4, 61.0, 78.1, 78.4, 102.5, 123.9, 130.2. 133.3, 153.9, 171.9.
