# Supplementary material for: N-carboxyacyl and N-α-aminoacyl derivatives of aminoaldehydes as shared substrates of plant aldehyde dehydrogenases 10 and 7
Source: Amino Acids. 2024 Aug 29;56(1):52. doi: 10.1007/s00726-024-03415-4 (PMC11362210; doi:10.1007/s00726-024-03415-4)
Supplement: Supplementary file 2 — Supplementary file2 (DOCX 308 KB) [file 726_2024_3415_MOESM2_ESM.docx]

***N*-carboxyacyl and *N*-α-aminoacyl derivatives of aminoaldehydes as shared substrates of plant aldehyde dehydrogenases 10 and 7**

**Michaela Masopustová^1^, Adam Goga^1^, Miroslav Soural^2^, Martina Kopečná^3^, and Marek Šebela^1^**

^1^Department of Biochemistry, ^2^Department of Organic Chemistry, and Department of Experimental Biology^3^, Faculty of Science, Palacký University, Olomouc, Czech Republic

**SUPPLEMENTARY FILE 2**

**Molecular mass data for novel aminoaldehydes (derivatives of dicarboxylic acids) including experimental MALDI-TOF MS results**

All these synthetic compounds were evaluated by direct measurements with the CHCA+CTAB matrix or measured with the same matrix after acid hydrolysis at elevated temperatures. The numbers in italics refer to partial hydrolysis of the acid-labile acetal group indicating loss of ethanol moiety/moieties.

| Compound with protecting groups (ester diethylacetal) | Theoretical molecular mass [Da] | Theoretical molecular mass of free acid-aldehyde [Da] | *m/z* detected by MALDI-TOF MS (direct measurements) | *m/z* detected by MALDI-TOF MS (after acid hydrolysis) |
| --- | --- | --- | --- | --- |
| **Ethyl MalAPAL diethylacetal** | 261 | 159 | *216* | 160 |
| **Ethyl SucAPAL diethylacetal** | 275 | 173 | *230* | 174 |
| **Ethyl GltAPAL diethylacetal** | 289 | 187 | *244* | 188 |
| **Ethyl MalABAL diethylacetal** | 275 | 173 | *184, 230* | 156 |
| **Ethyl SucABAL diethylacetal** | 289 | 187 | *198, 244* | 170 |
| **Ethyl GltABAL diethylacetal** | 303 | 201 | *212, 258* | 184 |

**Molecular mass data for novel aminoaldehydes (derivatives of Phe and Tyr) including experimental MALDI-TOF MS results**

All these synthetic compounds were evaluated by measurements with the CHCA+CTAB matrix after acid hydrolysis at elevated temperatures.

| Compound with protecting groups (Boc or Boc+tBu diethylacetals) | Theoretical molecular mass [Da] | Theoretical molecular mass of free acylated aldehyde [Da] | *m/z* detected by MALDI-TOF MS (after acid hydrolysis) |
| --- | --- | --- | --- |
| **Boc-PheAPAL diethylacetal** | 394 | 220 | 221 |
| **Boc-Tyr(tBu)-APAL diethylacetal** | 467 | 236 | 237 |
| **Boc-PheABAL diethylacetal** | 408 | 234 | 217, 235 |
| **Boc-Tyr(tBu)-APAL diethylacetal** | 481 | 250 | 233, 251 |

MALDI-TOF MS of *N*-carboxyacyl derivatives of APAL after the activation hydrolysis of their ester-acetals. From the top: MalAPAL, SucAPAL and GltAPAL. Spectra were measured with 1% (v/v) samples using CHCA matrix in the presence of CTAB according to Guo et al.,2002. The most significant CHCA matrix signals appeared at *m/z* 172 and 190 and were used for calibration.

Guo Z, Zhang Q, Zou H, Guo B, and Ni J (2002) A method for the analysis of low-mass molecules by MALDI-TOF mass spectrometry. *Anal. Chem*. **74**, 1637-1641. https:// doi/10.1021/ac010979m

MALDI-TOF MS of MalABAL after the activation hydrolysis of its ester-acetal. From the top: hydrolysis at 100 °C for 15 min, hydrolysis at 70 °C for 2 h. Spectra were measured with 6 mM solutions (calculated for the initial diethylacetal compound) using CHCA matrix in the presence of CTAB according to Guo et al.,2002. The most significant CHCA matrix signals appeared at *m/z* 172 and 190 and were used for calibration as well as the CTAB signal at *m/z* 284.

MALDI-TOF MS of PsAMADH reaction mixtures with GltAPAL and GltABAL. Spectra were measured with 1 mM solutions (calculated for the initial diethylacetal compound) using CHCA matrix in the presence of CTAB according to Guo et al.,2002. The most significant CHCA matrix signals appeared at *m/z* 172 and 190 and were used for calibration. Relative intensity is normalized to that of *m/z* 172.

**Relative reaction rates for native PsAMADH and recombinant ALDH7s with novel synthetic substrates (derivatives of dicarboxylic acids)**

Results were obtained by spectrophotometric measurements with 1 mM compounds in the reaction mixture. The reaction rates for the reference substrates APAL and AASAL were arbitrarily taken as 100 % for ALDH10 and ALDH7, respectively.

| Substrate | Relative rate with ALDH10 -PsAMADH  (in percentage) | Relative rate with PsALDH7  (in percentage) | Relative rate with ZmALDH7  (in percentage) |
| --- | --- | --- | --- |
| **APAL/AASAL*** | 100 | 100* | 100* |
| **MalAPAL** | 52 | 5.7 | 5.4 |
| **SucAPAL** | 110 | 1.6 | 1.1 |
| **GltAPAL** | 50 | 1.6 | 2.2 |
| **ABAL** | 65 | n.d. | n.d. |
| **MalABAL** | 40 | 6.2 | 5.9 |
| **SucABAL** | 52 | 1.8 | 2.8 |
| **GltABAL** | 56 | 2.1 | 2.8 |

**Relative reaction rates for native PsAMADH and recombinant ALDH7s with novel synthetic substrates (derivatives of Phe and Tyr)**

Results were obtained by spectrophotometric measurements with 1 mM compounds in the reaction mixture. The reaction rates for the reference substrates APAL and AASAL were arbitrarily taken as 100 % for ALDH10 and ALDH7, respectively.

| Substrate | Relative rate with ALDH10 -PsAMADH  (in percentage) | Relative rate with PsALDH7  (in percentage) | Relative rate with ZmALDH7  (in percentage) |
| --- | --- | --- | --- |
| **APAL/AASAL*** | 100 | 100* | 100* |
| **PheAPAL** | 5.7 | 11.3 | 9.0 |
| **PheABAL** | 4.1 | 4.5 | 1.5 |
| **TyrAPAL** | 3.2 | 11.8 | 8.6 |
| **TyrABAL** | 4.8 | 1.2 | 0.9 |

**Supplement to Fig. 3.**

**Saturation curves of native PsAMADH reactions with synthetic *N*-carboxyacyl** **aminoaldehydes derived from ABAL and reference substrates**. All measurements were conducted at pH 9.0 with the initial presence of 1 mmol·l^-1^ NAD^+^ and varying substrate concentrations. The depicted data were averaged from three independent measurements, as indicated by error bars. Individual substrates are represented by color coding, as explained at each graph.
